# Interleukin-6 Is a Crucial Factor in Shaping the Inflammatory Tumor Microenvironment in Ovarian Cancer and Determining Its Hot or Cold Nature with Diagnostic and Prognostic Utilities

**DOI:** 10.3390/cancers17101691

**Published:** 2025-05-17

**Authors:** Hina Amer, Katie L. Flanagan, Nirmala C. Kampan, Catherine Itsiopoulos, Clare L. Scott, Apriliana E. R. Kartikasari, Magdalena Plebanski

**Affiliations:** 1 School of Health and Biomedical Sciences, RMIT University, Bundoora, VIC 3082, Australia; 2 School of Medicine and Health Sciences, University of Tasmania, Launceston, TAS 7250, Australia; 3 Tasmanian Vaccine Trial Centre, Clifford Craig Foundation, Launceston General Hospital, Launceston, TAS 7250, Australia; 4 Department of Obstetrics and Gynecology, Faculty of Medicine, Universiti Kebangsaan Malaysia, Kuala Lumpur 56000, Malaysia; 5 The Walter and Eliza Hall Institute of Medical Research, Parkville, VIC 3052, Australia; 6 Faculty of Medicine, Dentistry, and Health Sciences, The University of Melbourne, Parkville, VIC 3052, Australia; 7 The Royal Women’s Hospital, Parkville, VIC 3052, Australia

**Keywords:** ovarian cancer, interleukin 6, diagnosis, prognosis, immune evasion, metastasis, drug resistance, inflammation, hot and cold tumors, tumor microenvironment, artificial intelligence, machine learning

## Abstract

Ovarian cancer (OC) remains the leading cause of cancer-related deaths among women, often diagnosed at advanced stages due to the lack of effective early diagnostic procedures. To reduce the high mortality rates in OC, reliable biomarkers are urgently needed, especially to detect OC at its earliest stage, predict specific drug responses, and monitor patients. The cytokine interleukin-6 (IL6) is associated with low survival rates, treatment resistance, and recurrence. In this review, we summarize the role of IL6 in inflammation and how IL6 contributes to ovarian tumorigenesis within the tumor microenvironment, influencing whether the tumor is subsequently classified as “hot” or “cold”. We further dissect the molecular and cellular mechanisms through which IL6 production and downstream signaling are regulated, to enhance our understanding of its involvement in OC development, as well as OC resistance to treatment. We highlight the potential of IL6 to be used as a reliable diagnostic biomarker to help detect OC at its earliest stage, and as a part of predictive and prognostic signatures to improve OC management. We further discuss ways to leverage artificial intelligence and machine learning to integrate IL6 into diverse biomarker-based strategies.

## Simple Summary

We review how interleukin-6 influences the ovarian tumor microenvironment, determining its “hot” or “cold” characteristics. We also examine the critical role of this cytokine in cancer progression, metastasis, and therapy resistance. Given the high mortality associated with this disease, there is a pressing need for reliable biomarkers. In this review, we emphasize the potential of interleukin-6 to enhance detection, predict drug responses, and assess prognostic outcomes.

## 1. Introduction

**Figure 1:**
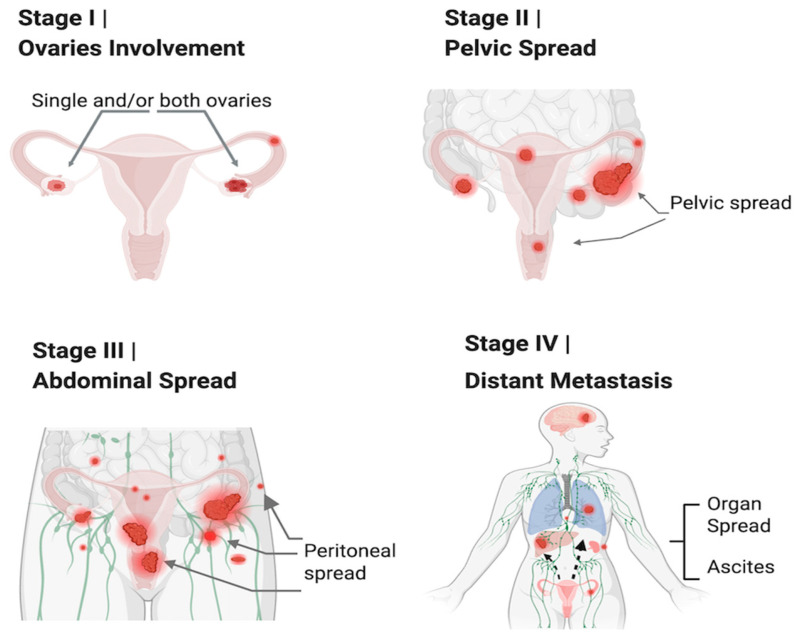
Stages of Ovarian Cancer: Four stages of Ovarian Cancer. Stage I: Confined to ovaries; Stage II: Cancer spread in pelvis; Stage III: Cancer spread in abdomen; Stage IV: Distinct metastasis. The red areas and arrows show local and distinct metastasis of Ovarian cancer.

Ovarian cancer (OC) is one of the most lethal gynecological malignancies, largely due to its diagnosis at advanced stages. The disease is classified into four stages: stage I (SI), stage II (SII), stage III (SIII), and stage IV (SIV), according to the FIGO (Federation of Gynecology and Obstetrics) system (Figure 1). Unfortunately, OC is often detected at later stages (SIII and SIV), which significantly increases the risk of morbidity and mortality [1]. Data from Cancer Australia shows that 2.4% of women in Australia were diagnosed with ovarian cancer in 2023. The chance of surviving for at least 5 years between 2015 and 2019 is only 49% [2]. Women with Breast Cancer Susceptibility Gene-1 (BRCA1) or BRCA2 mutations have a significantly higher risk, with a 27–44% chance of developing OC, compared to just 1% in the general population [3].

Given the poor prognosis and low survival rates associated with late-stage diagnosis of OC, understanding the molecular and immunological mechanisms driving its pathogenesis is crucial. Cancer survival and response to immunotherapy are strongly influenced by the presence or absence of active immune cells, which define the tumor as having a “hot” or “cold” tumor microenvironment (TME), respectively [4]. The “hot vs. cold” classification has been validated in several tumor types beyond OC. For instance, in melanoma and non-small cell lung carcinoma, “hot” tumors with high T cell infiltration show a favorable response with checkpoint inhibitor therapy [4,5,6]. In gynecological cancers such as endometrial and cervical cancer, a “hot” TME correlates with better prognosis and improved treatments, while cold tumors are related to poor outcomes and resistance [7]. Similarly, in high-grade serous ovarian cancer (HGSOC), which often exhibits an immunosuppressive “cold” TME, efforts to “heat up” the TME through cytokine modulation or combination therapies are being actively explored [8].

Interleukin-6 (IL6) is a key pro-inflammatory cytokine implicated in the initiation and progression of OC. It plays a significant role in promoting tumor invasion, metastasis, and therapeutic resistance. IL6 contributes to a “hot” TME by promoting T cell infiltration and activation in early inflammation but also drives a “cold” environment by sustaining chronic inflammation, expanding immunosuppressive cells, and impairing antigen presentation [9]. Moreover, the inflammatory microenvironment induced by IL6 has been shown to influence the tumor’s ability to switch between “cold” and “hot” phenotypes. which in turn affects its response to immunotherapy [10,11]. IL6’s involvement in these processes highlights its potential as a diagnostic, prognostic, and therapeutic biomarker.

This review aims to provide the current understanding of the role of IL6 in inflammation and how IL6 contributes to ovarian tumorigenesis, immune evasion, metastasis, and resistance to treatment. We will also delve into the molecular and cellular mechanisms by which IL6 drives the pathogenesis of OC, including its influence on determining whether the cancer adopts a “hot” or “cold” tumor profile. Identifying whether the tumor microenvironment (TME) is “hot” or “cold” can help guide the development of precision therapies and serve as a useful biomarker to predict how patients will respond to immunotherapy. Additionally, we will discuss the complex mechanisms by which IL6 affects tumor growth, metastasis, and therapy resistance. Finally, we will examine the potential of IL6 as a diagnostic tool, as well as its utility in predicting drug response and serving as a prognostic biomarker, with the goal of improving OC management.

## 2. Ovarian Cancer Immunogenicity: “Hot” or “Cold”

### 2.1. Tumor Microenvironment: “Hot” or “Cold”

**Figure 2:**
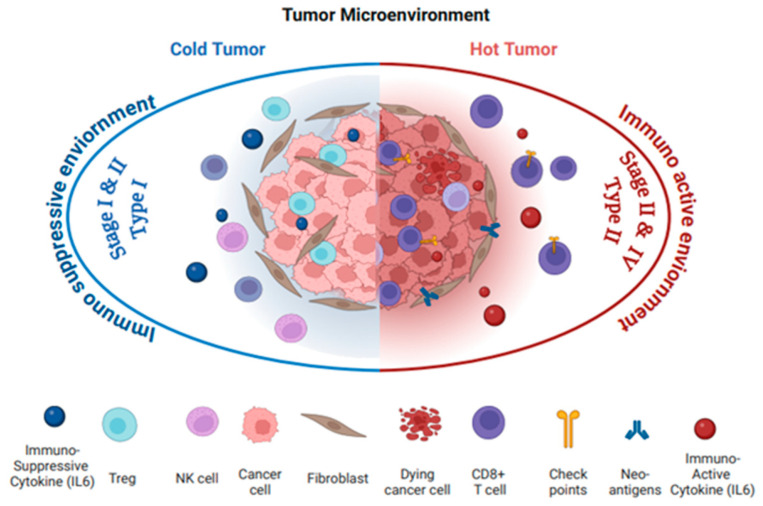
Hot and Cold Tumor microenvironment. LHS: “Cold” tumor microenvironment with less infiltration of immune cells; RHS: “Hot” and active tumor microenvironment with high immune infiltration; IL6 (Interleukin 6); Treg (T regulatory) cells; NK (Natural Killer) cells.

Tumor immunophenotypes classify tumors as “hot “or “cold”, which supports the development of personalized treatment strategies in cancer management. “Hot tumors” are characterized by highly tumorigenic, inflamed, and aggressive immune microenvironments, signaling that the immune system has recognized the tumor as a threat and activated a response to combat it (Figure 2). This robust immune reaction is essential for targeting and eliminating cancer cells. In the “hot” TME, the recruitment of pro-inflammatory immune cells is marked by elevated levels of interleukin-6 (IL6), tumor necrosis factor alpha (TNFα), interleukin-12 (IL12), and interferon gamma (INFγ). These cytokines further recruit and activate immune cells, particularly CD8^+^ cytotoxic T lymphocytes (CTLs) and natural killer (NK) cells, to attack the tumor. Additionally, there is an increased expression of checkpoint molecules, such as Programmed death-ligand-1 (PDL1) and cytotoxic T lymphocyte-associated protein-4 (CTLA4). The heightened immune activity in “hot” tumors makes them prime candidates for effective immunotherapy responses, including checkpoint inhibitors (such as anti-PDL1 and anti-CTLA4), which restore T cell function by blocking inhibitory pathways, and CAR (Chimeric Antigen Receptor) T cell therapy, which harnesses genetically engineered T cells to target and kill cancer cells [12,13].

In contrast, a “cold tumor” is less tumorigenic and is characterized by a lack of immune activity and minimal cell infiltration. “Cold” tumors are often dominated by immunosuppressive cytokines, such as IL6, IL10, and TGFβ, which suppress the recruitment and activation of immune cells, creating an environment that promotes tumor progression (Figure 2). These tumors have very few CTLs or NK cells in the TME, resulting in a largely inactive immune response. Low immunogenic “cold” tumors typically harbor immunosuppressive cells, like regulatory T cells (Tregs), myeloid-derived suppressor cells (MDSCs), and tumor-associated macrophages (TAMs), which contribute to immune evasion. “Cold” tumors lack key immune activation markers or checkpoint molecules, such as PDL1 and CTLA4, which further reduces their ability to respond to therapies and contribute to resistance against immunotherapy [12]. The low tumor mutational burden and poor antigen presentation make it difficult for the immune system to identify and target the cancer cells. As a result, “cold” tumors present significant challenges for immunotherapy compared to a “hot”, inflamed TME, as the immune system is less equipped to recognize and destroy the tumor [14].

### 2.2. OC Immunophenotype with Cancer Stages and Histological and Molecular Types

OC is generally considered a “cold” (low immunogenic) tumor; however, depending on the stage and histological subtype, it may exhibit features of a “hot” (high immunogenic) tumor phenotype [14,15]. Classifying OC by stages, histological types, and the dualistic model aids in defining its immunogenicity as “hot” and “cold” tumors, which can significantly help design personalized treatment strategies and reduce the cancer rate in women.

The OC staging is based on the tumor’s location and extent of spread, as classified by FIGO (Federation of Gynecology and Obstetrics), categorizing OC into four Stages (Figure 1). Stages “I” and “II” are “early stages”, where the cancer is confined to one or both ovaries and the pelvis, while Stages “III” and “IV” are “late stages”, marked by abdominal invasion and distinct metastasis, respectively [16]. OC is further divided into three main histological subtypes: the most common, epithelial ovarian cancer (EOC), which accounts for 90% of cases, and less common are ovarian germ-cell ovarian tumors (OGCTs) and sex cord-stromal tumors (SCSTs), which together make up about 10% of cases [17].

EOC, as the most common subtype, originates from the ovarian epithelial tissue and is further divided into four histological types: serous (80%), endometrioid (10%), mucinous (<3%), and clear cells (>5%) [18]. These subtypes can then be further recognized in a dualistic model approach that classifies EOC into two main subtypes: Type I and Type II (Table 1 and Table 2).

Type I encompasses less aggressive, genetically stable stage I cancers, predominantly “cold” tumors, characterized by low immune cell infiltration and immune exclusion. Their pathogenesis is mainly driven by distinct somatic mutations, with specific mutational profiles and association OC subtypes mentioned in Table 1 and Table 2. These include low-grade serous carcinoma (LGSC) (2% of all OC), low-grade endometrioid carcinoma (10%), mucinous carcinoma (2–3%), and clear cell carcinoma (5%), as well as rarer tumor types like Brenner and seromucinous tumor [19,20]. These cancers are often diagnosed at earlier stages (Stage I) and tend to maintain this immune-poor phenotype even as they progress to later stages (Stages III–IV) due to their slow growth and lack of immune activation. They evade immune surveillance through mechanisms involving cytokines like IL6, TGFβ, and the immune checkpoint molecules such as PDL1 and CTLA4, making them less responsive to immunotherapy [12].

On the other hand, Type II EOC is an aggressive and rapidly growing cancer that over 75% of patients are diagnosed with at advanced, often incurable stages (III and IV), with a 90% mortality rate [18]. The most common subtype within Type II is high-grade serous carcinoma (HGSC), which makes up about 75% of cases. Type II EOC is characterized by highly stable gene mutations, with BRCA and TP53 found in more than 35% and 95% of HGSC cases, respectively. The homologous recombination repair pathway is predominantly disrupted in Type II EOC, further fueling its aggressiveness [19,20]. Type II EOC, particularly HGSC, is generally “hot” tumors, with significant immune infiltration and activation. However, despite the immune response, these tumors still employ immune evasion strategies, such as the expression of PDL1 and other checkpoint inhibitors, which can hinder the effectiveness of immunotherapy [12].

**Table 1:** Ovarian Cancer Type I and involvement of Interleukin 6 in its pathogenesis.

Ovarian Cancer Subtype	Immune Phenotype and Hot/Cold Tumor	OC Stages	Genetic Mutations	Gene Expression	Epigenetic Changes	Signaling Pathways	Effect and Role of IL6
Endometrioid Carcinoma [21,22]	Cold Tumor: Low T cell and NK cell infiltration, poor immune response	I, II, III	PTEN, CTNNB1 (β-catenin), ARID1A	Estrogen receptor (ER) upregulation, β-catenin expression	Loss of ARID1A, DNA methylation changes	TGFβ pathway disruption, Wnt/β-catenin pathway, PI3K/AKT pathway	IL6 promotes tumor growth by activating JAK/STAT3 signaling, enhancing inflammation, survival, and resistance to apoptosis. It contributes to immune suppression and tumor progression.
Clear Cell Carcinoma [23,24]			ARID1A, PIK3CA	HIF1α overexpression, VEGF upregulation	DNA methylation, histone modifications	VEGF signaling, PI3K/AKT, HIF1α driven pathways, ARID1A-related chromatin remodeling	IL6 activates STAT3 and MAPK, promoting chemotherapy resistance, tumor progression, and immune evasion. It contributes to immune suppression and promotes angiogenesis.
Mucinous Carcinoma [25,26]			KRAS, PIK3CA	MUC1 overexpression	DNA methylation, histone modifications	EGFR pathway activation, MAPK pathway, PI3K/AKT pathway	IL6 activates the JAK/STAT3 pathway, promoting cell proliferation, survival, and migration. It enhances metastatic potential and contributes to immune evasion.
Low-Grade Serous Carcinoma [27]			BRAF, KRAS	Anti-apoptotic proteins (e.g., Bcl-2) upregulation	Epigenetic alterations in cell cycle regulation	MAPK/ERK pathway, PI3K/AKT pathway, BRAF/KRAS-related signaling	IL6 activates STAT3 signaling, contributing to tumor progression, chemotherapy resistance, and immune evasion. It enhances survival and promotes cell migration.

**Table 2:** Ovarian Cancer Type II and involvement of IL6 in its pathogenesis.

Ovarian Cancer Subtype	Immune Phenotype and Hot/Cold Tumor	OC Stages	Genetic Mutations	Gene Expression	Epigenetic Changes	Signaling Pathways	Effect and Role of IL6
High-Grade Serous Carcinoma (HGSC) [28,29]	Hot Tumor: PDL1 expression, increased T cell infiltration, higher cytokine levels (e.g., IL6, IL2, IFNγ)	III, IV	TP53, BRCA1/2, MYC over- expression	Cyclins and CDKs upregulated, MYC overexpression	DNA methylation in tumor suppressor genes (e.g., BRCA1)	PI3K/AKT/mTOR activation, Wnt/β-catenin signaling, MAPK pathway, p53-related pathways	IL6 activates STAT3 signaling, promoting cell proliferation, survival, metastasis, and immune evasion. It enhances angiogenesis and contributes to chemotherapy resistance.
High-Grade Endometrioid Carcinoma [30,31,32]		I, II, III	PTEN, CTNNB1 (β-catenin), ARID1A	Estrogen receptor (ER) upregulation, β-catenin expression	Loss of ARID1A, DNA methylation changes	Wnt/β-catenin pathway, PI3K/AKT pathway, TGFβ pathway disruption	IL6 promotes tumor growth by activating JAK/STAT3 signaling, enhancing inflammation, survival, and resistance to apoptosis. It can suppress the immune response and contribute to immune evasion.
BRCA1/2-Related Tumors [33,34]	Cold Tumor: Low T cell and NK cell infiltration, poor immune response	III, IV	BRCA1/2 mutations (loss of function)	RAD51 upregulation, compensatory DNA repair mechanism	Dysfunction in homologous recombination repair pathways	Fanconi anemia pathway disruption, sensitivity to PARP inhibitors and platinum chemotherapy	IL6 enhances tumor survival by activating JAK/STAT3, contributing to chemotherapy resistance, immune suppression, and tumor progression. It reduces immune cell infiltration.
Undifferentiated High-Grade Carcinoma [35]		III, IV	TP53 mutations, often no clear histologic differentiation	High cyclin D1 and CDK4 expression, varied gene expression	DNA methylation, chromatin remodeling, TP53 mutation- associated alterations	Activation of PI3K/AKT, MAPK, and aberrant cell cycle control pathways	IL6 activates STAT3 and MAPK, promoting chemotherapy resistance, tumor progression, and immune evasion. It contributes to immune suppression and supports metastasis.

### 2.3. IL6 Role in “OC Immunogenicity”

IL6 is a crucial mediator of the inflammatory response within the ovarian cancer tumor microenvironment (OC-TME). Its role is multifaceted, either promoting immune activation and a “hot” TME or, conversely, driving immune exhaustion and developing a suppressive “cold” TME [36]. It exerts its effects by activating signaling pathways such as the Janus kinase/signal transducer and activator of transcription (JAK/STAT) pathway, which influences not only immune cells but also non-immune cells within the TME.

In Type I EOC, which is generally associated with the “cold” TME, IL6 contributes to immune evasion by promoting an immunosuppressive microenvironment, often through the upregulation of regulatory T cells (Tregs) and the inhibition of cytotoxic T cells (CTLs) activity. Several signaling pathways are commonly altered in Type 1 EOC, including mitogen-activated protein kinase (MAPK) pathways, such as KRAS or BRAF; Phosphoinositide 3-kinase (PI3K) pathways; the mammalian target of rapamycin (mTOR) pathway; Wnt signaling pathways (Wnt)/β-catenin; and serine-threonine kinase 1(AKT) pathways, which are linked to IL6-mediated signaling [20,37,38].

In Type II EOC, the “hot” immunophenotype of OC, particularly HGSC, IL6 is aggressively involved in driving immune evasion and tumor progression, such as upregulating immune checkpoint molecules, including PD1 and CTLA4. The IL6-driven chronic inflammatory environment can contribute to immune exhaustion, despite the presence of active immune cells [39]. Its role extends to facilitating metastasis and ascites formation, both of which are hallmarks of late-stage ovarian cancer [40]. The elevated levels of IL6 in the TME stimulate the production of pro-inflammatory cytokines and chemokines, further promoting cancer cell survival, proliferation, and migration to distant organs (Table 1 and Table 2, Figure 1 and Figure 2) [36,41,42].

The ovarian cancer tumor microenvironment (OC-TME) is typically considered “cold” due to the lack of immune activation and limited immune cell infiltration. However, IL6 blockade can “heat up” the TME and shift a “cold” TME to a “hot” one by fine-tuning the immune system towards a more anti-tumor phenotype, thereby enhancing the effectiveness of targeted immunotherapies [11,43]. Inhibiting IL6 reduces the excessive inflammatory signaling, lowering the production of inflammatory mediators like T helper 17 (Th17) cells, which promote a shift to more immunosuppressive Tregs [44]. This reduction in Tregs can help alleviate immune tolerance and re-establish immune cell activity against the tumor. IL6 inhibition can also suppress B-cell activation and antibody production, further decreasing tumor-promoting effects driven by IL6 [42].

Furthermore, even in a “hot” TME, IL6 blockade can shift the environment towards effective immune elimination [11]. Elevated IL6 has been linked to chemotherapy resistance in ovarian cancer by activating pro-survival signaling pathways, such as JAK/STAT3 and PI3K/AKT, which enable tumor cells to withstand chemotherapy-induced stress. Inhibiting IL6 signaling can disrupt these survival pathways, potentially sensitizing tumor cells to chemotherapy and reducing resistance [36,41,42]. Moreover, IL6 blockade not only alters immune cell differentiation but can also improve the efficacy of immunotherapies such as checkpoint inhibitors and CAR-T cell therapy, while mitigating chemotherapy resistance. Thus, combining IL6 inhibitors, such as tocilizumab, with immunotherapies and chemotherapy could not only boost their effectiveness but also help reduce the aggressiveness of the OC-TME, offering a promising strategy for improving patient outcomes [36,41,42].

## 3. IL6 in Inflammation and Cancer

IL6 is a critical mediator that immediately orchestrates the alert response signals in emergencies, including cancer, by signaling through various immune cells, such as macrophages and monocytes, that patrol the body [45]. Understanding the regulation and activation of IL6, particularly in the context of cancer, could provide valuable insights for improving diagnostic procedures and developing targeted treatments.

The expression of IL6 is stimulated by multiple stimulants, including toll-like receptors (TLRs) ligands, TNF, IL1, reactive oxygen species (ROS), and zinc (Zn) [45]. The regulation of IL6 expression is inherently tied to its structural configuration, characterized by a tetra-α- helical bundle topology, presented as a ribbon structure [46]. The distinctive arrangement of four α-helices provides a larger surface area, essential for IL6’s interaction with the receptor and the activation of subsequent signaling pathways.

**Figure 3:**
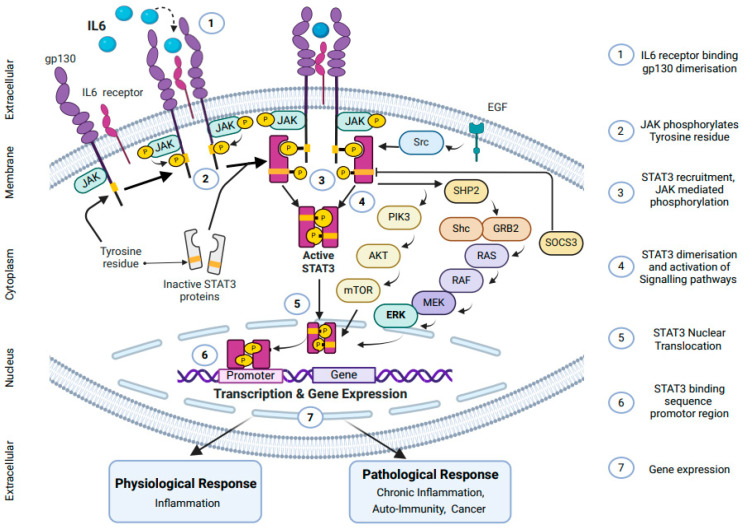
IL6 acts as a key upstream activator by binding to its receptor complex, IL6R and gp130, initiating multiple intracellular signaling pathways such as JAK/STAT3, MAPK/ERK, and PI3K/AKT. These pathways, shown as arrows within the cell, lead to nuclear translocation and transcriptional activation of the target gene involved in inflammation, survival, and immune regulation.; JAK (Janus kinase); STAT (signal transducer and activator of transcription); gp130 (glycoprotein 130); EGF (epidermal growth factor); Src (protooncogene tyrosine protein kinase Src); PIK3 (phosphatidylinositol 3 kinase); AKT (protein kinase B); mTOR (mammalian target of rapamycin); Ras (rat sarcoma protein); Raf (rapidly accelerated fibrosarcoma); MEK (mitogen-activated protein kinase kinase); MAPK (mitogen-activated protein kinase); SOCS3 (suppressor of cytokine signaling-3); SHP2 (protein tyrosine phosphatase, non-receptor type); Shc (Src homology 2 domain containing transforming protein C).

IL6 can recognize and bind to two distinct receptors and initiate two separate signal transmission pathways (Figure 3) [47]. One receptor type is a membrane-bound IL6 receptor (mbIL6R), an 80 kD α-chain protein known as CD126, gp80, or IL6α. These receptors are expressed in a limited range of cell types, including certain immune cells such as T cells, neutrophils, monocytes, megakaryocytes, hepatocytes, and endothelial cells [48]. Upon binding with these receptors, IL6 forms a complex with IL6R (IL6/IL6R), activates gp130, and initiates signaling through anti-inflammatory “classical signal pathways”. This activation leads to various targeted physiological functions, such as stimulating amyloid-beta precursor protein (APP) production in response to inflammation [49]. This trans-signaling IL6 activation divergently acts as a pro-inflammatory activity of IL6, associated with chronic inflammation and tumorigenesis [50].

The second type of receptor for IL6 is found in circulation and referred to as the soluble IL6 receptor (sIL6R). These receptors are 50–55 kD protein molecules lacking cytoplasmic extension and are present at plasma levels of approximately 25–35 ng/mL in healthy individuals [51]. The formation of sIL6R occurs through alternating mRNA splicing (10% of cases) and predominantly by proteolytic cleavage and ectodermal shedding (90% of cases) of membrane-bound IL6 receptors (mbIL6Rs) via proteases such as a disintegrin and metalloproteinase-10 (ADAM10) or ADAM17 from the ADAM gene family [52]. The binding of IL6 to sIL6R forms the soluble receptor complex (IL6/sIL6R), allowing IL6 to exert its effects in any cell with transmembrane signal transducers, gp130 β-chain receptors. This interaction triggers the pro-inflammatory “trans-signaling pathway” (34). Studies show that this soluble complex plays a significant role in hematopoiesis, neuronal survival, osteoclast proliferation, stimulation, and activation of endothelial cells, as well as smooth muscles [53,54,55].

The IL6 molecule has three conserved epitopes, namely, I, II, and III, specific for binding with IL6Rα, gp130, and the IgG domain of gp130, respectively. The ubiquitous expression of gp130 in nearly all body cells accounts for the diversity of IL6-elicited responses [56]. IL6 initially binds to IL6R, followed by binding to the gp130 β-receptors, leading to gp130 homodimerization and downstream signaling [56]. Two molecules of each activated complex IL6/IL6R/gp130 form a high-affinity hexameric structure, followed by gp130 dimerization, which leads to initiation of intracellular signaling transduction through various pathways, including the JAK/STAT3 pathway and MAPK pathway [56].

Soluble gp130 (sgp130) has been shown as an additional regulator for IL6/IL6R signaling generated by alternative splicing [56,57]. It acts as a natural antagonist, neutralizing soluble IL6 receptors complex and inhibiting trans-signaling [56,57]. Elevated plasma levels of sgp130 serve as a potent negative regulator of the trans-signaling pathway mediated by sIL6R, thereby controlling the pro-inflammatory effect of IL6 [58].

### 3.1. IL6 Signaling in Inflammation

Inflammation is an immediate sterile response triggered by the activated innate immune cells, including macrophages, neutrophils, dendritic cells (DCs), eosinophils, basophils, mast cells, and natural killer (NK) cells. This orchestrated activation aims to eliminate pathogens and facilitate tissue healing. The localized immune cells release chemical mediators such as cytokines and chemokines and present antigens to initiate antigen-specific T cell-mediated and B cell (humoral) responses within the adaptive immune system [59]. The ideal outcome of the inflammatory process is the sequential engagement of innate and adaptive immune systems, resulting in physiological healing and restoration of hemostatic function [60].

Inflammatory processes elicited by the external infectious stimuli release pathogen-associated molecular patterns (PAMPS) such as LPS from the bacterial wall. Additionally, endogenous molecules known as damage-associated molecular patterns (DAMPs) are released from stressed, damaged, or dying cells, including those from cancerous, autoimmune, or hypoxic tissues. Acute inflammation begins within minutes to hours of tissue injury, triggered when pattern recognition receptors (PRRs) detect PAMPs and DAMPs near the site of damage [61,62]. These PRRs are present on immune cells (such as macrophages, mast cells, and dendritic cells) that reside in tissues or circulate in the bloodstream, as well as on non-immune cells (like epithelial cells and fibroblasts) within the tissue matrix. Furthermore, they are expressed on the surface of leukocytes (neutrophils and monocytes) that migrate to the injury site [62,63]. Once PRRs identify antigens, immune cells trigger a cascade of vascular and cellular inflammatory events, including increased vascular permeability, recruitment of leukocytes, and activation of inflammatory mediators [61].

Local release of the pro-inflammatory cytokines like IL6, IL1α/β, IL8, IL16, IL17, TNFα/β, and tumor growth factor-beta (TGFβ) accelerates the innate response and serves as a bridge to activate the adaptive immune system [64]. Moreover, the chemokines, vasoactive amines (serotonin and histamine), eicosanoids, including prostaglandins (PGs), thromboxane (TXs), leukotrienes (LTs), lipoxins (LXs), and exotoxins, act independently or synergistically to establish and maintain the acute response of the innate inflammatory phase [37,65]. PGs promote vasodilation and enhance the effect of other mediators, like histamine, kinin, and serotonin [37]. Once the inflammatory response has successfully cleared pathogens, resolution mediators such as resolvins (RvD1), purines (adenosine), gaseous mediators (H_2_O and CO_2_), neuromodulators, various proteins and peptides, and lipoxins (LX4) activate anti-inflammatory signals. These mediators help return tissue to a state of homeostasis, thereby resolving the inflammation [60].

The acute inflammatory mediators triggered by PAMPs activate PRRs and activate the NFκB (Nuclear factor kappa-light-chain-enhancer of activated B cells) signaling pathway, which plays a crucial role in regulating both inflammatory and immune responses [61]. NFκB interacts with transforming growth factor-β-activated kinase 1 (TAK1), which is associated with the regulatory units of TAK-binding protein-1 (TAB1) and TAB2. TABs bind to polyubiquitin chains, activating TAK1, and the IKK kinase complex. This leads to the phosphorylation of IκBα, followed by the activation of the NFκB pathway. NFκB is a significant inducer of pro-inflammatory cytokines, including IL1β, IL6, IL12, and TNFα, and chemokines expression in various immune system cells [66] IL1β and TNFα amplify IL6 production, which not only further influences the synthesis of IL1β and TNFα, but also acts as a warning signal in response to DAMPs, which in turn activates TLR9 and initiates additional NFκB signaling, amplifying the inflammatory response [48].

IL6 is recognized as a multifunctional, pleiotropic cytokine with a wide range of roles in inflammation, immune response, hematopoiesis, and tissue maintenance [48]. Initially, IL6 was categorized based on its various biological activities, with different names reflecting its distinct functions. It was known as Cytotoxic T-lymphocyte differentiation factor (CDF) and B-cell stimulatory factor-2 (BSF-2) due to its ability to stimulate and differentiate T and B cells, respectively. It was also referred to as hepatocyte stimulatory factor (HSF) for its role in inducing the production of acute phase proteins (APPs) from hepatocytes, and HGF (Hybridoma growth factor) for its effect on plasma and myeloma fusion cells. Additionally, it was known as Interferon beta-2 (INFβ2) due to its antiviral activity and was associated with hematopoietic factors, as it plays a role in stimulating the growth of hematopoietic cells [48]. However, later, in 1986, the cloning of BSF-2 cDNA revealed that all these functional variations are attributed to the same cytokine [67].

IL6 produced locally in the inflammatory environment enters the bloodstream and stimulates hepatocytes to release acute phase reactants (APRs) [68]. APRs promote the synthesis of proteins such as C-reactive protein (CRP), haptoglobins, serum amyloid-A (SAA), and α1-antichymotrypsin, while decreasing the production of albumin, transferrin, and fibronectin [69]. These proteins play key roles in the acute phase of inflammation, helping to initiate anti-inflammatory responses. For example, CRP activates the complement system through the classic C1q pathway and mannose-binding protein (MBP), which aids in phagocytosis and promotes tissue repair [70]. Circulating IL6 also acts on the bone marrow to promote megakaryocyte maturation and increases platelet production, facilitating clotting [71]. Moreover, IL6 induces hypoferremia (reduced iron levels) by inhibiting the iron transporter ferroportin 1 in the intestine and triggers the expression of the zinc transporter-14 (ZIP14) in liver cells, leading to hypozincemia (reduced zinc levels) [72,73,74]. Together, these factors contribute to the resolution of inflammation.

IL6 plays a unique role among major cytokines, contributing to the acute inflammatory response and activating both cellular and humoral immune responses by promoting the activation and differentiation of T and B cells [59]. It synergistically interacts with other mediators to perform essential physiological functions. IL6 also enhances hematopoiesis, which gives rise to multiple blood cell lineages [75]. IL6 also promotes the differentiation of cytotoxic CD8^+^ T cells and, in combination with TGFβ, facilitates the upregulation of Th17 cells from naïve CD4^+^ cells while inhibiting Tregs [76].

Regarding humoral immunity, IL6 plays a crucial role by enhancing the production of IL21 and promoting the differentiation of T helper (Th) cells and activated B cells. This ultimately leads to the production of immunoglobulins and antibodies, which can contribute to hypergammaglobulinemia and the formation of autoantibodies [77]. Additionally, IL6 can activate osteoclasts via RANKL (Receptor Activator of NFκB Ligand) signaling, leading to bone resorption and potentially contributing to osteoporosis [78]. It also stimulates the expression of vascular endothelial growth factor (VEGF), enhancing vascular permeability and promoting angiogenesis [79].

### 3.2. IL6 Signaling in Cancer

In physiological states, IL6 plasma levels are maintained at low levels (<8 (0.2–7.8) pg/mL) to regulate basal hemostasis [80]. However, in response to infections, autoimmune diseases, or tumors, IL6 levels can surge dramatically [80]. One of IL6’s critical roles is in immune tolerance. It helps modulate the balance between Th17 cells and Tregs, and an imbalance in this regulation can lead to autoimmune diseases and chronic inflammation [76]. Elevated IL6 levels also emerge in the aging population, especially in post-menopausal women, where it contributes to immune dysfunction and a favorable environment for cancer development [61]. Excessive IL6 levels have also been associated with the development of ascites in OC [81].

The idea that inflammation plays a role in cancer was first proposed by Rudolf Virchow in 1863 [82]. Virchow suggested that immune cells, particularly lymphocytes, in the tumor microenvironment (TME) contribute to tumor growth and metastasis. This was later supported by the work of scientists like Busch, Fehleison, and William Coley, who demonstrated that infection-induced immune responses could cause tumor regression. Later, in 1976, Morales et al. found that the bacterium BCG (Bacillus Calmette-Gue’rin) was an effective treatment for bladder cancer [83]. In 1889, Paget proposed the “seed and soil theory” to explain cancer metastasis, suggesting that cancer cells develop a tumor microenvironment (TME) through a favorable interaction of cancer cells (seed) with the host environment (soil) [84].

The theories led to understanding the crucial role of the immune system in identifying and encountering cancer cells through immune surveillance, a concept initially proposed by Ehrlich in the mid-20th century and later refined by Burnet and Thomas [85]. It has been revised and is now recognized as “immune editing”, consisting of three “E” phases, “elimination, equilibrium, and escape” [86]. These phases are crucial in defining tumors based on immunogenicity, classifying them as “hot tumors” or “cold tumors”. IL6 is one of the most critical cytokines shaping OC-TME through immunoediting of tumors, influencing whether they are classified as “hot” or “cold” [6]. This is evident in various studies where IL6 levels have been documented to be incredibly high in late stages (SIII and SIV) and ascitic fluid of OC [87].

The first phase of immunoediting is the “elimination phase”, in which innate and adapted immunity work together to identify and clear up the cancer cells. Highly immunogenic tumors are more likely to be successively cleared due to their ability to attract immune cells and trigger robust immune responses. IL6 influences the activation of innate immune cells (such as macrophages and dendritic cells) and adaptive immune cells (such as T cells), enhancing the anti-tumor response and supporting the elimination of tumor cells. However, its overproduction can also contribute to an inflammatory microenvironment, potentially driving immune dysfunction [36,86]. The remaining or reappearing cancer cells enter the second phase, the “equilibrium phase”, during which the immune system controls net tumor growth by putting selective pressure without completely eradicating it. The tumor may not be clinically evident, but the phase is associated with tumors transitioning between “hot” and “cold” tumor phenotypes.

IL6 influences this phase by modulating the immune TME, providing survival of certain cells and allowing them to adapt and persist. IL6 signaling pathways aid in maintaining tumor dormancy, potentially keeping the tumor clinically undetectable while still exerting immune pressure. However, the mutational alteration through genetic and epigenetic changes in cancer may lead to the third phase, the “escape phase”, which is the most critical hallmark of cancer. In this phase, the cancer cells escape immune recognition by reducing their immunogenicity, leading to uncontrolled proliferation and eventual clinical presentation of a tumor [86]. The immune escape phase is highly related to the downregulation of antigen presentation, recruitment of immunosuppressive cells (such as Tregs and MDSC), and secretion of inhibitory cytokines. IL6 plays a significant role in promoting the “cold tumor” phenotype through immune suppression by the expansion of certain immune cells (such as Tregs) and inhibition of others (such as CTL) [86].

This generalized tumor editing concept is evident at different phases in various cancers [6]. OC is often referred to as “cold” tumors, as they typically bypass the initial two phases (elimination and equilibrium during their dormant stage, usually SI and SII) and are diagnosed at the late, inevitable stages (SIII and SIV) [88]. At this point, the coolness of its microenvironment creates challenges for therapeutic management.

Understanding OC-TME is crucial for optimizing management strategies. IL6 influences all three “E” phases of immunoediting, playing a key role in modulating the tumor’s immunophenotype, i.e., “hot” and “cold”. Therefore, it is vital to investigate the role of IL6 in OC-TME and its effect on non-immune and immune (innate and adaptive) cells within OC-TME. Understanding the OC-TME can enhance diagnosis, prognosis, and prediction of therapeutic responses for precision in OC treatments, including targeted therapies and combination with traditional and other immunotherapies of OC.

## 4. IL6 in the Tumor Microenvironment of Ovarian Cancer

**Figure 4:**
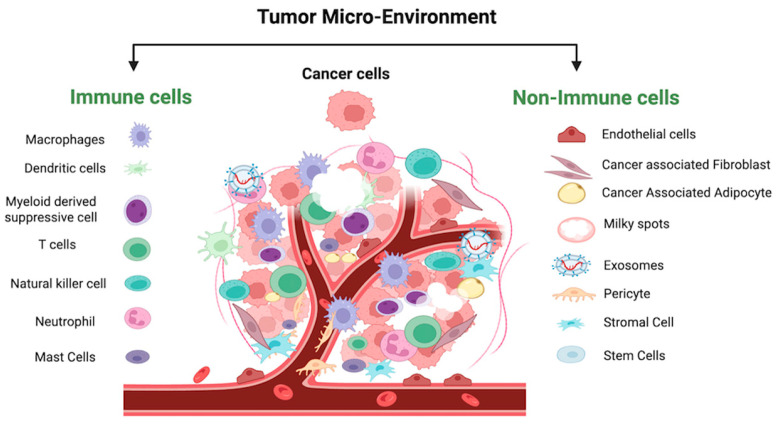
Immune and non-immune cells in the tumor microenvironment: The immune cells include macrophages, dendritic cells, myeloid-derived suppressive cells, natural killer cells, and neutrophils. The non-immune cells include endothelial cells, cancer-associated fibroblasts, cancer-associated adipocytes, pericytes, stromal cells, stem cells, exosomes, and milky spots.

The OC-TME is a highly specialized and adaptive cellular ecosystem that evolves to support tumor growth and metastasis (Figure 4) [88]. It acts as a self-sustaining entity, developing its own blood supply, meeting its nutritional demands, and facilitating disease progression. The OC-TME is rich in components such as secretory cytokines, chemokines, integrins, matrix metalloproteinases (MMPs), and exosomes, which are all integral to the environment’s complexity and ability to support tumor survival and spread [89,90].

As the OC-TME develops, intricate interactions between immune and non-immune cells foster an environment that not only supports tumor growth but also enables immune evasion. These interactions culminate in creating an immunosuppressive microenvironment, which promotes the survival of tumor cells, aids in metastasis, and helps tumor cells escape immune surveillance. This process is often facilitated by specialized immune cells known as “milky spots”, which contribute to the formation of malignant ascites in advanced ovarian cancer, a hallmark of aggressive disease [91]. These factors collectively enable the tumor to thrive, even in the face of immune defense.

The OC-TME is populated by a variety of cells, including both non-immune and immune types. Non-immune cells, such as endothelial cells, cancer-associated fibroblasts (CAFs), cancer stem cells (CSCs), and cancer-associated adipocytes (CAAs), invade the tumor microenvironment and help shape its immune-suppressive landscape. Immune cells, such as tumor-associated macrophages (TAMs), dendritic cells (DCs), lymphocytes (T and B cells), and granulocytes (e.g., neutrophils, natural killer (NK) cells), are also present but often become dysfunctional or suppressed, contributing to the tumor’s ability to evade immune detection and attack [36,90]. The aggressiveness of this interplay between immune and non-immune cells intensifies as ovarian cancer progresses to later stages, which correlates with an increase in morbidity and mortality among patients. Understanding the role of IL6 in manipulating both immune and non-immune cells within the OC-TME is therefore essential for improving therapeutic strategies and predicting their effectiveness.

### 4.1. IL6 Crosstalk with Non-Immune Cells in OC-TME

The extracellular matrix (ECM) in the OC-TME provides vital structural and functional support to tumor cells, blood vessels, and surrounding tissues. It helps fulfill the tumor’s metabolic needs while also facilitating communication between tumor and non-malignant cells. Within the ECM of the OC-TME, a variety of non-immune cells reside, including cancer-associated fibroblasts (CAFs), endothelial cells, pericytes, stromal cells, stellate cells, and cancer-associated adipocytes (CAAs), all of which play a crucial role in supporting tumor growth and metastasis.

Cancer-associated fibroblasts (CAFs), the key components of the TME, are associated with poor prognosis in OC [92]. CAFs can originate from quiescent fibroblasts, undergo epithelial-mesenchymal transition (EMT), or derive from bone marrow mesenchymal stem cells or the endothelial-to-mesenchymal transition (EndMT). These transformations are driven by various cytokines such as IL6 and IL1, miRNAs, ECM remodeling, and autocrine signaling [92]. In OC, four subtypes of CAFs (CAF-S1 to CAF-S4) have been identified, with CAF-S1 and CAF-S4 representing activated forms of CAFs, and CAF-S2 and CAF-S3 representing quiescent forms. CAF-S1, in particular, can be further subdivided into inflammatory CAFs (iCAFs) and myofibroblast CAFs (myCAFs). These activated CAFs are instrumental in driving the inflammatory and immune-suppressive status of the TME, which exacerbates tumor progression [92]. IL6 plays a critical role in activating CAFs, particularly through the JAK/STAT signaling pathway. iCAFs, identified in the ascitic fluid of OC patients, secrete IL6 and chemokines such as CXCL12 and CXCL2, further promoting the inflammatory immunosuppressive microenvironment [92]. MyCAFs, on the other hand, create a dense ECM that facilitates tumorigenesis [92]. In addition, other signaling pathways, such as the Hedgehog (Hh), NFκB, MAPK, platelet-derived growth factor (PDGF), and focal adhesion kinase (FAK), contribute to CAF activation, with IL6 acting as a key driver in these processes [93]. Activated CAFs are also linked to metabolic distress within the TME, including increased glycolysis, glucose depletion, and the development of an acidic environment, all of which hinder the functioning of tumor suppressors and promote tumor cell survival.

Tumor-associated OC endothelial cells within the OC-TME exhibit increased expression of IL6 receptor (IL6R), and upon activation by IL6, they induce the expression of vascular endothelial growth factor (VEGF), which drives angiogenesis [94]. Pericytes which surround endothelial cells, contribute to angiogenesis and help establish a stem cell reservoir in the TME, further aiding tumor growth and metastasis [95]. IL6 levels contribute to defective pericyte coverage, which can be improved with anti-IL6 therapy in xerograft models [95]. The IL6/IL6R/gp130 complex activates signaling pathways via STAT3 and MAPK activation, which not only drive angiogenesis but also contribute to tumor progression [94].

Stromal cells in the OC-TME, under the influence of pro-inflammatory mediators like prostaglandin E-2 (PGE2) and cyclooxygenase-2 (COX-2), also express and produce stromal-derived factor-1 (SDF1), which, upon binding to its receptor CXCR4 on cancer cells, induces angiogenesis and immune cells recruitment [96]. This further supports the tumor’s ability to grow and metastasize.

Cancer-associated adipocytes (CAAs) are another key player in the OC-TME. These adipocytes promote tumor progression, neovascularization, and metastasis by secreting adipokines such as IL6 and leptin. This creates a highly immunosuppressive environment that favors cancer cell survival and spread [97]. Additionally, IL6 signaling in adipocytes, in turn, induces the release of leptin, which impacts various metabolic processes, including liver metabolism and the function of pancreatic tissues. IL6 also stimulates the p38 MAPK pathway in mesenchymal stem cells, enhancing adipogenesis and contributing to the tumor-supportive environment [98].

Exosomes are the nano-sized extracellular vesicles released by OC cells that are abundant within the ECM of OC-TME and ascitic fluid [99]. These exosomes play a significant role in OC progression and metastasis by facilitating intercellular communication and influencing the surrounding cellular environment [89]. These exosomes carry bioactive molecules responsible for the stemness and development of the pre-metastatic niche [100]. These OC cell-derived exosomes in OC-TME have been found to induce IL6 secretion and can exert their effects by activating the STAT3 signaling pathway, promoting cell proliferation, survival, and migration [89]. This IL6-driven signaling cascade not only contributes to tumor cell growth but also enhances the immune escape mechanisms within the TME, affecting both immune and non-immune cells within OC-TME.

### 4.2. IL6 Crosstalk with Immune Cells in OC-TME

#### 4.2.1. IL6 and Innate Immune Cells

**Figure 5:**
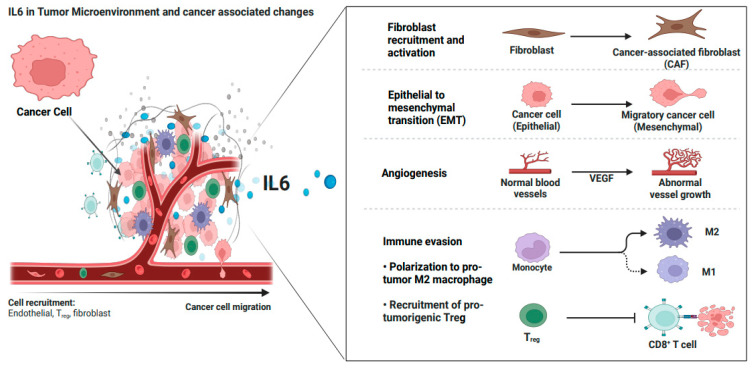
IL6 action on various cells in the tumor microenvironment and resulting cancer-associated changes: IL6 promotes fibroblast transition to cancer-associated fibroblasts (CAFs); it induces epithelial-to-mesenchymal cells transition in tumor cells; stimulates angiogenesis by upregulating VEGF (vascular endothelial growth factor) expression; drives the polarization of monocytes to protumor M2 macrophages; and enhances the recruitment of immunosuppressive protumorigenic regulatory T cells (Tregs).

The ovarian cancer tumor microenvironment (OC-TME) is characterized by aberrant, immature, and activated innate immune cells that play a significant role in promoting immune evasion, tumor progression, and metastasis. These innate immune cells include myeloid-derived suppressor cells (MDSCs), tumor-associated macrophages (TAMs), tumor-associated neutrophils (TANs), mast cells, dendritic cells (DCs), and natural killer (NK) cells, among others. IL6 is a central mediator that modulates the function and behavior of these immune cells, contributing to a pro-tumorigenic environment that facilitates cancer cell survival and dissemination (Figure 5).

Myeloid-derived suppressor cells (MDSCs) are a heterogeneous group of immature myeloid cells that are pathologically activated and prevent an effective immune response against tumors [101] They include mononuclear (M-MDSC) and polymorphonuclear (P-MDSC) subsets, each with distinct surface markers and immunosuppressive functions. M-MDSCs (CD33^+^ CD14^+^ HLA-DR^−^/lowCD15^−^) and P-MDSCs (CD33^+^ CD14^−^ CD15^+^) are known to produce high levels of reactive oxygen species (ROS), arginase, and nitric oxide (NO), which inhibit T cell activity and induce apoptosis, ultimately leading to immune suppression in the TME [101]. These cells also impair the function of NK cells, thereby contributing further to immune evasion [101]. IL6 plays a pivotal role in the activation and differentiation of MDSCs, along with other pro-inflammatory factors such as IL1β and prostaglandin E2 (PGE2) [102]. High levels of IL6 in OC are associated with poor clinical outcomes, as they lead to the accumulation of immunosuppressive MDSCs in the TME, inhibiting the function of both T cells and NK cells and promoting metastasis [102]. Recent studies have classified ovarian cancer patients into two categories based on IL6R expression and MDSC cells. Those with low IL6R expression exhibited less mature myeloid cell infiltration and better overall survival (OS), whereas those with high IL6 levels, high IL6R expression, and high infiltration of tumor-associated CD163^+^ myeloid cells were associated with poor clinical outcomes [103].

Dendritic Cells (DCs) are critical for initiating anti-tumor immune responses by presenting tumor antigens to T cells. However, in the OC-TME, IL6 modulates DC differentiation and impairs the expression of key molecules such as MHCII and CD86, which are necessary for effective T cell activation [104]. IL6 also suppresses the migration of DCs by suppressing the expression of CCR7 on DCs, a chemokine receptor involved in DC migration [105]. Additionally, DCs in the OC-TME secrete IL6 in a paracrine manner, helping maintain their immature, immunosuppressive state and further hindering anti-tumor immune responses [105].

Tumor-associated macrophages (TAMs) are among the most abundant immune cells in the OC-TME and are heavily involved in promoting tumor progression, angiogenesis, and metastasis [106]. TAMs can exist in two phenotypic states: M1-like, which are generally anti-tumorigenic, and M2-like, which are pro-tumorigenic. In OC, IL6 skews TAMs towards the M2-like phenotype, enhancing their ability to secrete immunosuppressive factors. IL6 also promotes the recruitment, activation, and survival of M2-TAMs in the TME, thereby contributing to an immune-suppressive and pro-tumorigenic environment. M2-TAMs also facilitate angiogenesis, forming new blood vessels that provide nutrients and oxygen to the growing tumor. Additionally, M2-TAMs participate in tissue remodeling processes that support tumor invasion and metastasis. By interacting with tumor cells, M2-TAMs can promote their survival and growth [106]. These functions of TAMs collectively contribute to the aggressive nature of OC in the tumor microenvironment, which is responsible for progression, metastasis, and therapy resistance in OC.

Natural killer (NK) cells are crucial for the innate immune response to tumors due to their ability to kill tumor cells directly and mediate antibody-dependent cellular cytotoxicity (ADCC), a process where NK cells destroy target cells coated with antibodies by binding to the Fc portion of the antibody using their CD16 receptor [13]. However, NK cell activity in the OC-TME is often impaired, as they show reduced cytotoxic activity and are less effective in controlling tumor growth [107]. Studies show that overexposure of CD15 and B7-H6 ligands present on the cancer cells to their activating receptors DNAX accessory molecule-1 (DNAM-1) and Natural Killer Protein-30 (NKp30) leads to NK impairment [108]. Additionally, CA125 induced alterations in the NK cell phenotype, leading to downregulated CD16 on NK cells, impaired Fc receptor-based ligation, compromised antibody-dependent cellular cytotoxicity (ADCC), and suppressed NK cell activity [109]. IL6 has been shown to downregulate the expression of activating receptors on NK cells, such as NKG2D and NKp30, which impairs their ability to recognize and kill tumor cells. Additionally, IL6 induces the expression of SHP-2, a phosphatase that inhibits NK cell activation, further dampening their cytotoxic potential [110]. While there is limited infiltration of NK cells within the primary ovarian tumor mass, an elevated presence of peritoneal fluid NK cells has paradoxically correlated with unfavorable outcomes in EOC patients, despite indications that higher levels of NK cells in TME predict a favorable prognosis [111]

Tumor-associated Neutrophils (TANs) Neutrophils are typically involved in the initial inflammatory response to infection and injury by eliminating pathogens through phagocytosis and creating neutrophilic extracellular traps (NETs) that immobilize and neutralize them [112]. However, they can be recruited to the TME and adopt a tumor-promoting phenotype as TANs, which correlates with poor prognosis [113]. IL6 plays a role in enhancing neutrophilic trafficking and activity in physiological and pathological conditions. Studies indicate that the interplay of IL6 and the STAT3 pathway regulates the dynamics of neutrophil migration and influences the generation of TANs [113]. Furthermore, IL6, in conjunction with other mediators such as IFNβ and GM-CSF, influences the activation and longevity of TANs by initiating an anti-apoptotic response [113]. In TME, TANs act primarily by generating reactive oxygen species (ROS) and other factors that inhibit T cell function, leading to T cell immunosuppression and tumor growth [114]. A high neutrophil-to-lymphocyte ratio (NLR) has been identified as a poor prognostic factor in ovarian cancer, with increased IL6 levels being associated with a higher NLR and worse clinical outcomes [115].

#### 4.2.2. IL6 and Adaptive Immune Cells—T Cells

Adaptive immune cells, particularly T cells, play crucial roles in the immune response against cancers [13]. Among T cells, there are two major subsets: T helper (CD4^+^) cells and cytotoxic (CD8^+^) T cells. Cytotoxic CD8^+^ T cells, along with certain subsets of CD4^+^ T cells (such as Th1, Th2, and cytotoxic CD4^+^ cells), are particularly associated with potent anti-tumor immunity [13]. (DCs) are responsible for presenting antigens to T cells through major histocompatibility complex (MHC) Class I and Class II molecules, which are recognized by CD8^+^ and CD4^+^ T cells, respectively [13]. This interaction initiates T cell priming through T cell receptor (TCR) signaling. Upon antigen interaction, CD8^+^ T cells become effector CD8^+^ T cells or Cytotoxic CD8^+^ T cells (CTLs), while CD4^+^ produce cytokines, further potentiating CD8^+^ differentiation [13]. TCR activation also requires the binding of DC ligands CD70 and CD80-CD86, with costimulatory receptors on T cells, CD27 and CD28, respectively [13].

Chemokine ligands, such as CXCL9 and CXCL10, released by DCs, attract CTLs expressing CXCR3, aiding their migration and infiltration into TME [13]. NK cells also play a role by interacting with DCs, further activating CD4^+^ T cells and enhancing cytokine production, subsequently activating CTLs [13]. Once activated, CTLs target and kill tumor cells by perforating the cell membrane with perforin, releasing granzymes A and B to induce DNA fragmentation [13]. Additionally, the Fas ligand pathway, activated by CTLs, triggers apoptosis in tumor cells through caspase activation and cytochrome-c release [116].

Studies have shown that increased infiltration of CD8^+^ T cells correlates with improved survival in ovarian cancer (OC) patients. CD8^+^ T cells are effective anti-tumor mediators, and their higher presence within ovarian TME is associated with improved outcomes [117,118]. The presence of CD3^+^ and cytotoxic CD8^+^ T cells within the OC-TME is associated with improved prognosis and better survival rates following chemotherapy-induced remission [119,120,121].

Regulatory T cells (Tregs) are crucial for maintaining immune homeostasis by preventing inappropriate immune activation in a resting state and facilitating the resolution of immune responses at inflammatory sites. However, in cancer, they are often implicated in immune suppression, which facilitates tumor growth [122]. The immunostimulatory functions of CTLs, NK cells, and M1-type macrophages are counteracted by interactions with Tregs in the TME [122]. For instance, by inhibiting the activity of CTLs, Tregs suppress the elimination of cancer cells [122]. Tregs in the TME produce adenosine, a byproduct of ATP (Adenosine triphosphate) breakdown, which promotes the secretion of TGFβ from MDSCs, alters NK cell function, and further potentiates immunosuppressive activity in TME [122,123]. Furthermore, Tregs also secrete immunosuppressive cytokines, such as TGFβ and IL10, which induce the production of immature DC (CD14^+^ CD1a^−^), incapable of effectively presenting antigens to T cells [122,123].

Elevated Treg levels in the TME are associated with poor therapeutic response and prognosis in cancer patients [124]. Tregs expressing TNFR2 contribute to an immunosuppressive environment and are linked to poor outcomes [40]. TNFR2-associated upregulation of CTLA4 and glycoprotein A repetitions predominant (GARP) contributes to chemoresistance [125]. Additionally, Tregs lead to the expression of PDL1 on ovarian cancer cells, an immune checkpoint that further impedes anti-tumor immunity [126].

The balance between CD8^+^ cytotoxic T cells and Tregs in the TME significantly influences cancer immune surveillance and serves as an important prognostic factor for ovarian cancer. A higher ratio of CD8^+^ T cells to Tregs is generally associated with better immune control of the tumor. In contrast, an increased presence of Tregs suppresses CD8^+^ T cell activity, allowing cancer cells to evade immune surveillance and promoting tumor growth and metastasis. This CD8^+^/Treg ratio is increasingly considered a key determinant of patient survival in ovarian cancer [118,127].

Elevated IL6 levels in the TME can disrupt the balance between CD8^+^ T cells and Tregs, promoting tumor growth and reducing the effectiveness of chemotherapy [128]. High IL6 levels correlate with a transcriptionally quiescent state in CTLs, marked by reduced expression of key activation and effector molecules, such as IFN-γ, TNFα, and granzyme B. This results in impaired cytotoxic activity against tumor cells [128]. High IL6 levels in cancer patients correlate with a transcriptionally quiescent state in CTLs, marked by reduced expression of genes associated with activation and effector function [39]. Indeed, CTLs in patients with low IL6 exhibit heightened effector characteristics and functional activity [39].

In OC, ascitic fluid contains high levels of IL6 and leukemia inhibitory factor (LIF), which trigger the formation of TAM-like cells from monocytes, further promoting tumor progression and metastasis [129]. TAMs in the OC-TME produce TGFβ, which inhibits the activity of infiltrating CD8^+^ T cells, exacerbating immune suppression and contributing to tumor growth [130]. IL6 also sustains anti-apoptotic factors, aiding T cell survival in the TME [131].

IL6 plays a critical role in the activation of Tregs within the TME and mediates the upregulation of TNFR2 expression in Tregs in ascites, which correlates with poor survival in OC patients [132]. Blocking IL6 in ascites results in reduced TNFR2 expression and improved patient outcomes [132].

IL6 further upregulates immune checkpoint molecules such as PD1 and CTLA4 on CTLs, promoting tumor immune evasion [128]. Tumor cells often exploit these immune escape mechanisms by expressing PDL1 and CTLA4, suppressing further anti-tumor immunity [13,122,128]. IL6 signaling contributes to the upregulation of these immune checkpoint molecules, thereby limiting the efficacy of therapies designed to block them. Consequently, IL6-mediated immune dysregulation represents a critical barrier to effective cancer immunotherapy and serves as a target for therapeutic interventions aimed to restore immune function.

## 5. Orchestrated Role of IL6 in Ovarian Cancer Development, Metastasis, and Recurrence

IL6 is a key cytokine that closely correlates with the tumor size in OC, with its levels significantly elevated in the advanced stages of the disease [87,133]. In patients with extensive neoplastic effusions and ascites, IL6 further hallmarks as a major contributor to disease progression, complicating therapy response, and decreased overall survival of OC patients [133]. This has been verified by various studies, which report that IL6 levels are markedly higher in malignant cystic and ascitic fluids of OC patients compared to those with benign ovarian tumors [87]. In this discussion, we will explore the coordinated role of IL6 in the development, metastasis, recurrence, and metabolic changes associated with ovarian cancer (OC). Understanding this pathway will offer valuable insights into how IL6 can be considered in OC detection and how it may influence predictions of patient outcomes.

### 5.1. IL6 in OC Development and Progression

In cancer cells, IL6 promotes cell proliferation by both accelerating the cell cycle and preventing apoptosis. Via the JAK/STAT3 or NFκB pathways, IL6 upregulates the expression of the cell cycle-promoting proteins, including cyclin D1, D2, B1, and c-MYC. IL6 also downregulates the negative cell cycle regulators, including P21 and p27, thus facilitating the progression of the cell cycle and promoting cell proliferation. In apoptosis, IL6 upregulates the expression of anti-apoptotic proteins such as Bcl-2, Mcl-1, Bcl-xL, and survivin, via activating various signaling pathways, such as STAT3, NFκB, via JAK/STAT, NFκB, Phosphoinositide-3 Kinase/Protein Kinase B (P13K/AKT), and Mitogen-Activated Protein Kinase/Extracellular signal-regulated Kinase (MAPK/ERK) [134,135,136]. The anti-apoptotic proteins inhibit pro-apoptotic Bax, Bak, and Bim activation and thus prevent the downstream pathways, which are cytochrome-c release from mitochondria, leading to a caspase-dependent pathway of apoptosis, or by the release of apoptosis-inducing factors (AIF), leading to a caspase-independent pathway of apoptosis [137]. Caspases are critical enzymes that, on activation, cleave various cellular proteins and are responsible for the cell’s characteristic apoptotic appearance, such as membrane blebbing, chromatin condensation, and nuclear fragmentation [138].

IL6 involvement in OC development is not only promoting cell proliferation and inhibiting apoptosis. It also induces the generation of cancer stem cells (CSCs). CSCs are highly tumorigenic due to their ability to self-renew, differentiate, and proliferate. In OC, IL6 via STAT3 promotes the expression of CD44, a transmembrane glycoprotein associated with cancer cell stemness. CD44 binds to hyaluronan (HA) and other extracellular matrix (ECM) components to promote CSC stemness that leads to EMT stimulation, metastasis, and poor prognosis in OC patients [139]. Additionally, IL6 via the JAK/STAT pathway upregulates the expression of SMG1, a kinase enzyme. SMG1 is associated with lower immune infiltration and poor survival in several cancers, and it plays a role in initiating NMD (nonsense-mediated mRNA decay) [140]. NMD is a surveillance mechanism that degrades RNA transcripts containing premature stop codons, thus reducing the expression of tumor-specific frameshift-derived neoantigens and contributing to immune evasion [141].

In the TME, IL6 is required to promote angiogenesis, the formation of new blood vessels from existing ones, which is critical for tumor survival, providing essential pathways for a continuous supply of nutrients and the removal of waste products to sustain their rapid growth and survival. Angiogenesis is regulated by a delicate balance of pro-angiogenic and anti-angiogenic factors. Key angiogenic stimulators include VEGF, PDGF, PGE2, FGF2, TGFα and TGFβ, TNFα, cadherins, integrins, and EphB4/ephrin-B2, while inhibitors such as interferons, endostatin, angiopoietin-1 and -2, thrombospodin-1, and others, prevent excessive angiogenesis [142]. IL6 via the JAK/STAT3 pathway enhances the expression of hypoxia-inducible factor 1-alpha (HIF1α) under hypoxic and acidic conditions at tumor sites, leading to increased VEGF-A expression, which drives neovascularization and angiogenesis [143]. Furthermore, in OC, IL6 accumulation stimulates the production of metalloproteinases, particularly MMP9, facilitating cancer cell invasion and the degradation of the extracellular basement membrane [144]. MMP9 in turn activates VEGF and basic fibroblast growth factor (FGF2), supporting endothelial cell survival and growth. Fibronectin, another key factor involved in neovascularization and a negative prognostic indicator for OC, is also induced by IL6 [145].

### 5.2. IL6 Promotes OC Invasiveness and Formation of Ascitic Fluid

Epithelial-to-mesenchymal transfer (EMT) is a tightly regulated physiological process involved in wound healing, embryogenesis, and ovarian development. In OC, IL6 secreted by TAMs in the TME induces aberrant EMT, transforming epithelial cells into a mesenchymal phenotype. This shift enhances the production of mesenchymal proteins, such as vimentin and cadherin, and is linked to increased proliferation and invasiveness of OC cells [146]. The IL6-induced JAK/STAT3 signaling pathway elevates the expression of MMP9 in OC cells, degrading the extracellular matrix (ECM) and creating pathways for tumor invasion and metastasis [147]. MMP9, a zinc-dependent metalloproteinase, specifically breaks down type IV collagen in basement membranes. Increased levels of MMP9 in OC are associated with poor prognosis and higher recurrence rates [147]. IL6-induced Snail overexpression is also associated with increased transcription, expression, and activity of MMP2 in epithelial cells, causing degradation of the basement membrane and facilitating the successful invasion and metastasis of tumor cells [148]. Indeed, decreased invasiveness and metastasis were observed when MMP2 and MMP9 were downregulated [149].

Fibronectin, an ECM protein, promotes cell adhesion and migration. Elevated levels of fibronectin in the stromal tissue and peritoneal fluid of OC patients correlate with advanced tumor stages, greater invasiveness, metastasis, and poor DFS and OS [150]. In vivo studies show that IL6 facilitates fibronectin synthesis, and experiments in knockout mice reveal reduced invasiveness and metastasis of SKOV3ip1 cells when fibronectin is absent [151].

The “cadherin switch”, from E-cadherin to N-cadherin, is a key feature in many cancers, including OC [152]. Downregulation of E-cadherin and upregulation of N-cadherin are associated with increased invasiveness and poorer survival outcomes in OC patients [153]. E-cadherin is responsible for tight cell-to-cell contact through stable cadherin junctions and impacts the regulation of tissue homeostasis via various pathways, which maintains epithelial linings. N-cadherin promotes cell survival and migration by stabilizing the fibroblast growth factor receptor (FGFR) and activating the MAPK/ERK pathway [154]. Furthermore, IL6-induced JAK/STAT, MAPK, P13K/AKT, and NFκB pathways also lead to overexpression of EMT-transcription factor ZEB1 (zinc finger E-box binding homobox 1), Snail, Slug, and Twist [155]. These factors suppress CDH1 (cadherin 1) expression, which, in turn, hinders the production of transmembrane glycoprotein epithelial cadherin (E-cadherin) protein at cell-adherent junctions [156]. Loss of E-cadherin results in the weakening of cell-to-cell adhesion, and subsequent detachment of the epithelial cells from the underlying basement membrane leads to increased invasiveness of OC cells from tumor sites and is related to poor prognosis by upregulating expression of specific adhesion molecules (such as α5-integrin and CD44) that mediate cancer cell adhesions to mesothelial cells lining the abdominal cavity [157].

Other signaling pathways involved in the expression of the EMT phenotype are Wnt/β signaling, Hedgehog signaling, TGFβ signaling, Notch pathway, MAPK pathway, P13K pathway, NFκB pathway, Src signaling, and various growth factor signaling [155]. These signaling pathways also have a prominent role in promoting EMT in OC. Various studies have shown that elevated levels of IL6 are involved the expression and activation of various transcription factors responsible for EMT stimulation and the development of invasive and metastatic traits [155]. Additionally, IL6-mediated signaling pathways also downregulate endothelial tight junction proteins, such as occludin and claudin, and weaken the basement membrane of OC thereby facilitating the development of EMT and increasing invasiveness [155].

OC cells invade and metastasize rapidly through a series of complex steps. Typically, during metastasis, EMT-induced ECM degradation allows cancer cells to detach from the primary tumor site [158]. These cells then travel through the blood, lymphatic system, and peritoneal fluid to reach secondary tissues. Unlike many other cancers, OC’s most aggressive and common route of metastasis is trans-coelomic, with detached cancer cells easily migrating from the primary site to the peritoneal cavity [159]. Subsequently, OC cells release factors in an autocrine and paracrine manner, increasing the shedding of malignant cells into the peritoneum, allowing the tumor to spread to the omentum and the peritoneum (both parietal and visceral), eventually spreading to other organs [159]. OC can also directly disseminate into lymphatics and then enter the circulation to spread further to other organs [159].

The development of malignant ascites is one of the hallmarks of OC and is associated with a significantly poor prognosis, with a 5-year survival rate of only 5%, compared to 45% in stage III/IV OC [160]. OC cells can detach from the original site after ECM degradation [160]. As ovaries are closely aligned to the peritoneal cavity, the exfoliated cancer cells drop into the nearby naturally flowing peritoneal fluid current [160]. These malignant cells can float independently in the peritoneal/ascitic fluid or form spheroid clusters without solid scaffolds. In the fluid, OC cells can evade the immune system by secreting cytokines and increasing complement resistance [160]. Ultimately, these malignant cells implant on the omental mesothelium, following the “seed and soil” theory of metastasis [160].

In ascitic fluid, IL6 levels are markedly elevated (>2000 pg/mL) and correlate with rapid progression, worse prognosis, and shorter duration of disease-free survival [87]. High IL6 and TNFα levels in ascitic fluid from primary post-surgical patients have been linked to worse PFS and OS [161]. Elevated IL6 in ascitic fluid further suppresses the anti-tumoral immune response and contributes to the tumor progression [162]. The presence of higher volumes of ascitic fluid also presents significant challenges for the delivery and effectiveness of therapies [162].

### 5.3. IL6 Induces Treatment Resistance and Recurrence

The OC cell lines, such as SKOV3, C13, A2780, and ES2, showed increased STAT3 levels after cisplatin treatment and are associated with suppressing the effect of anti-apoptotic proteins like survivin, Bcl-2, and Bcl-xL [163]. In OC animal models, IL6 upregulates HIF1α, promoting resistance to cisplatin [143].

Activation of STAT3 also increases the expression of miR21a, and its overexpression can downregulate PTEN, a key regulator of the PI3K/Akt oncogenic pathway. This results in the proliferation of ovarian cancer (OC) cells and contributes to cisplatin resistance [164]. Additionally, therapy-induced inflammation plays a role in chemoresistance, with studies showing that platinum-based chemotherapy induces the oncogenic long non-coding RNA HOTAIR, which elevates IL6 levels and further promotes resistance to treatment [165].

Taxane-based therapies can similarly trigger the upregulation of functional IL6 production by activating several signaling pathways, such as JNK and TLR4, leading to increased chemoresistance [166]. In OVCAR3 cell lines, paclitaxel resistance has been linked to CAF-induced IL6 expression and the activation of JAK/STAT3 signaling [167]. Furthermore, the EGFR/ERK/NFκB and EGFR/PI3K/NFκB pathways play crucial roles in cancer cell proliferation and invasion, which in turn increase IL6 and IL6R expression in chemo-resistant ovarian cancer cells [168].

CSCs also contribute significantly to post-treatment resistance, driving tumor relapse by regenerating OC cells from remaining cancer cells. In CSCs, IL6 stimulates the expression of CD44^+^ through the JAK/STAT3 pathway, leading to the formation of a STAT3/Nanog complex. This complex upregulates multidrug resistance markers such as MDR1, YAP1, and IGF2BP3, contributing to therapeutic resistance [139]. Furthermore, high levels of IL6 and TNFα in OC patients can promote pre-treatment ascites development, increase chemoresistance, and result in shorter OS [161].

In OC-TME, elevated IL6 levels upregulate the expression of PDL1 and CTLA4 in cancer cells, which inhibits T cell activity and serves to escape the immune response [13]. IL6-induced immune evasion leads to rapid proliferation, metastasis, and resistance to chemotherapy. In OC patients, immune evasion is a key factor in the development of malignant ascites, which is associated with poor survival outcomes [169].

### 5.4. IL6 Worsens the Metabolic Status of OC Patients

IL6 significantly contributes to metabolic disturbances in OC, playing a central role in reprogramming metabolism. This disruption can lead to complications, including anemia, thrombocytosis, and immune suppression. In normal cells, energy is primarily produced through mitochondrial oxidative phosphorylation of glucose, generating ATP, NADH, and FADH_2_. Additionally, glucose is metabolized through the pentose phosphate pathway, which also helps neutralize toxic metabolic byproducts, such as the reactive oxygen species (ROS). 

In cancer cells, rapid growth increases the resting energy expenditure (REE) and basal metabolic rate (BMR). To meet these heightened energy demands, cancer cells rely on aerobic glycolysis, also known as the Warburg effect, where glucose is rapidly broken down into lactate to generate energy, despite the sufficient availability of oxygen. IL6 through STAT3 can further enhance this process by activating signaling pathways that promote glycolytic enzymes and glucose uptake, essentially “boosting” the Warburg effect [170]. For instance, IL6 through STAT3 activation inhibits pyruvate dehydrogenase activity by upregulating pyruvate dehydrogenase kinase. As a result, pyruvate is reduced to lactate instead of acetyl-CoA, which is required in mitochondrial oxidative phosphorylation. The excess lactate is transported to the liver, where it enters the Cori cycle to form glucose, a process that consumes significant energy and contributes to cancer-related weight loss [170].

In advanced OC stages, IL6 secreted by CAFs interacts with IL6R on OC cells, leading to the overexpression of hexokinase 2 (HK2). HK2 catalyzes the first irreversible step of glycolysis, the conversion of glucose to glucose-6-phosphate, thereby promoting the Warburg effect [170]. HK2 also regulates stemness properties of cancer cells by upregulating key stemness genes such as NANOG, SRY-Box 9 (SOX9), CD117, octamer-binding transcription factor 4 (OCT4), and Krüppel-like factor 4 (KLF4) [171]. Notably, studies have shown that neutralizing IL6 or IL6R antibodies can reduce CAF-induced expression of HK2, emphasizing the important role of IL6 in promoting cancer cell growth.

IL6 contributes to anemia by stimulating hepatocytes to upregulate hepcidin transcription through activation of the JAK/STAT3 pathway, which acts as a negative regulator of iron absorption in the duodenum and iron release from macrophages [73,172,173]. In the OC genetic model, levels of iron efflux pump receptor ferroprotein (FNP) increased, while levels of iron importer receptors, transferrin (TRF), decreased. This led to increased intracellular iron accumulation, which is associated with invasion, growth, and peritoneal metastasis of cancer cells [174].

Iron chelation and FPN induction have been shown to reduce IL6 mRNA and deactivate the STAT3 pathway, lowering intracellular iron levels and inhibiting OC tumor cell proliferation and metastasis. Additionally, elevated IL6 levels stimulate ROS production, which affects kidney function and reduces erythropoietin production, contributing to anemia [175].

IL6 activation of STAT3 affects pancreatic β cells, impairing insulin signaling and reducing insulin synthesis and release [176]. This results in insulin resistance, altered glucose uptake, and increased muscle proteolysis, weakening muscle mass [176]. IL6 diminishes triglyceride uptake in adipose tissue, leading to hypertriglyceridemia and hypercholesterolemia [177]. IL6’s impact extends to the brain, where it is involved in the development of “sickness responses” such as depression, mood disturbances, nausea, anorexia, and cachexia in OC patients. These symptoms are often linked to dysregulated hypothalamic–pituitary–adrenal (HPA) axis signaling [178]. IL6 also increases diurnal cortisol levels, which exacerbate vegetative depression; however, these symptoms improve in post-remission patients when IL6 and cortisol levels are lowered [179].

In low-energy situations, the central nervous system signals to activate glucose pathways to restore energy balance. However, elevated IL6 leads to increased release of corticotropin-releasing factor and promotes anorexigenic pathways while inhibiting orexigenic mediators such as neuropeptide Y (NPY) and agouti-related peptide (AgRP) [179]. This disruption of food intake regulation results in decreased appetite, weight loss, anemia, and reduced body fat [179]. These IL6-induced metabolic alterations lower energy levels and are associated with decreased leptin levels, a key metabolic biomarker that links immune responses with metabolic changes. Leptin levels are inversely correlated with IL6, inflammatory responses, and prognosis in OC [82,180]. Moreover, IL6 plays a crucial role in maintaining energy metabolism within the tumor microenvironment (TME) by impairing the ketogenic response, further exacerbating metabolic disturbances [181].

### 5.5. IL6 Effect on PGCCs in OC

Polyploid giant cancer cells (PGCCs) are increasingly recognized as key drivers of metastasis, recurrence, and drug resistance [182]. PGCCs arise in response to hypoxic conditions or chemotherapeutic stress and are characterized by large cells with multinucleation and irregular or enlarged nuclei. These PGCCs emerge in OC, particularly with high-grade serous Ovarian cancer (HGSOC), in response to cytotoxic agents like cisplatin or paclitaxel [183]. These cells can enter a dormant-like state and later generate progeny through a unique cell division known as neosis or budding [184]. The resultant progeny exhibit stem-like properties, fueling the tumor’s regenerative potential, contributing to tumor recurrence, chemoresistance, and heterogeneity [182].

IL6 plays a critical role in the stability and maintenance of PGCC cells in OC. Elevated IL6 levels activate STAT3 and NFκB pathways, promoting PGCC survival [185]. IL6 also enhances oxidative stress tolerance and DNA repair mechanisms, enabling the persistence of damaged polypoid cells [186]. Furthermore, IL6 supports the stem-like phenotype of PGCC-derived progeny and converts fibroblasts into GPR77^+^/CD10^+^ CAFs via collagen and VEGF upregulation [185]. The GPR77^+^/CD10^+^ CAFs are known to support chemoresistance and cancer stem cells in OC. These CAFs contribute to chemoresistance and support CSCs by creating a niche rich in IL6 and IL8, which foster a pro-inflammatory environment that enhances CSC survival, drives drug resistance, and facilitates tumor progression [185]. Thus, IL6 is involved not only in the induction of PGCCs in response to chemotherapy but also in continued proliferation and the support of making the elimination of OC cells more challenging.

## 6. IL6 and Current OC Management

The therapeutic management of OC involves a combination of surgery, chemotherapy, and targeted therapies. Additionally, classifying tumors as “hot” or “cold” based on their immune response helps define their immunophenotype, which can significantly aid in tailoring personalized immunotherapy for OC. Below, we highlight the current conventional therapies, targeted treatments, and immunotherapies for ovarian cancer (OC), as well as the effect of adding IL6 blockade, each of which plays a crucial role in optimizing patient outcomes.

### 6.1. OC Management with Current and Targeted Therapies

The therapeutic management of ovarian cancer has advanced, with ongoing research focused on reducing morbidity and improving patient survival. The standard treatment protocol typically involves cytoreductive surgery, which is followed by biopsy-based FIGO staging. Primary debulking surgery (PDS), including bilateral salpingo-oophorectomy, hysterectomy, and omentectomy, aims to leave residual tumor nodules smaller than 1 cm [187]. Despite these efforts, residual tumor tissue remains a major challenge due to its potential for aggressive inflammatory activity and disease recurrence [187].

To prevent further tumor growth from any remaining cells after suboptimal debulking, a combination of chemotherapy and radiation therapy is typically administered post-surgery. The optimal first-line treatment for ovarian cancer currently involves a combination therapy using platinum (carboplatin) and paclitaxel [188]. Platinum analogues (cisplatin and carboplatin) kill cancer cells by crosslinking with their DNA, while paclitaxel stabilizes microtubules, inhibiting cell division.

**Figure 6:**
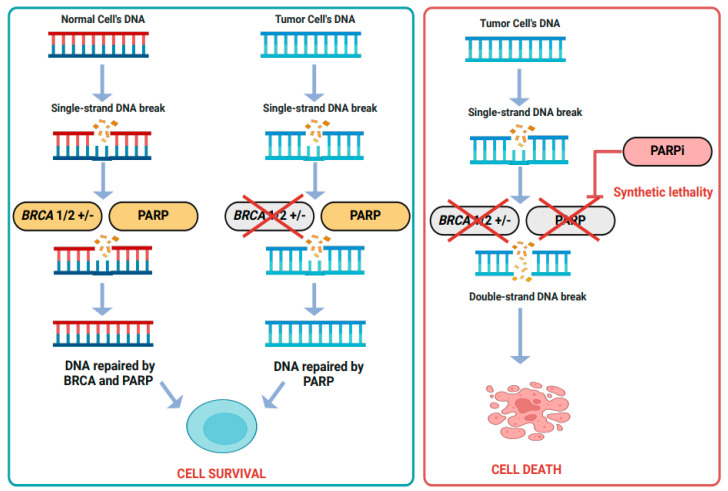
Targeted therapy with PARPi: PARP repairs single-stranded DNA breaks, and BRCA repairs double-stranded DNA breaks by the Homologous recombination process, ensuring the integrity of the genome. PARP inhibitors exploit the vulnerability of BRCA-mutated cells by blocking an alternative repair pathway, leading to synthetic lethality in Ovarian cancer; PARPi (poly ADP-ribose polymerase inhibitor); BRCA (breast cancer gene).

Targeted therapies play a crucial role in the management of OC, either as maintenance therapy in combination with first-line treatment or in cases of recurrence. OC is characterized by impaired DNA repair through homologous recombination (HR) deficiency (HRD) (Figure 6). In healthy cells, HR is primarily mediated by BRCA1 and BRCA2 proteins, along with PARP (Poly ADP-ribose Polymerase) enzymes [189]. PARP inhibitors (PARPi), such as Olaparib, exploit BRCA mutation by blocking single-strand DNA repair, inducing synthetic lethality [189]. Olaparib is widely used as a first-line maintenance therapy for advanced-stage OC patients who have shown partial or complete responses to platinum-based chemotherapy [189].

Bevacizumab, an anti-VEGF (vascular endothelial growth factor) antibody, is another targeted therapy that inhibits angiogenesis in OC-TME. This drug is typically administered in combination with other therapies or as maintenance therapy for patients with residual tumors larger than 1.0 cm, and it has shown improved outcomes in terms of PFS [190]. In preclinical animal models, VEGF inhibition has been shown to reduce tumor growth and ascites [191]. Olaparib, as a maintenance monotherapy or in combination with bevacizumab, has significantly improved PFS and OS in Phase III trials (SOLO-1 and PAOLA-1) in BRCA mutant patients, though no benefit in PFS is seen in cases that are homologous recombination deficient (HDR)-negative [189,192,193].

### 6.2. Low-Dose Cyclophosphamide Improves OC Recurrence and Relapses

Cyclophosphamide is a well-established chemotherapeutic drug that was initially administered intravenously before the current standard first-line therapy, which consists of platinum-based regimens and paclitaxel [194]. However, recent studies suggest that low-dose oral cyclophosphamide, when administered in a metronomic fashion, may offer benefits in improving PFS and OS in patients with recurrent or relapsed ovarian cancer [195].

Cyclophosphamide is both an immunosuppressive and neoplastic agent that works by crosslinking DNA and RNA within and between DNA strands through the release of its active alkylating agent. This mechanism inhibits protein synthesis and suppresses Tregs, particularly within the OC-TME. This effect may help trigger an anti-tumor response in the OC-TME [196]. The use of oral cyclophosphamide in a metronomic regimen for OC is still an area of ongoing research, and further studies are needed to fully assess its efficacy and safety.

### 6.3. Immunotherapies for OC

Immunotherapies have emerged as a promising approach for managing OC by harnessing the patient’s immune system to target and combat cancer cells within the OC-TME. These therapies work by stimulating the immune system to recognize and attack cancer cells, or by providing synthetic immune components, such as antibodies and cytokines, to boost the immune response [13,36]. There are various types of immunotherapies, including monoclonal antibodies, immune checkpoint inhibitors, cancer vaccines, and adoptive cell therapy. The primary goal of these treatments is to enhance the immune system’s ability to identify, target, and ultimately eliminate cancer cells. Immunotherapies aim to overcome immune evasion by cancer cells, increase immune responses, and improve the body’s ability to fight cancer, ultimately leading to tumor regression and elimination while minimizing damage to healthy tissues.

Immune checkpoints are inhibitory pathways in the immune system that play a key role in maintaining self-tolerance and regulating immune responses in peripheral tissues, helping to minimize collateral damage to healthy tissues. However, tumors can exploit these immune checkpoint pathways to evade attacks from T cells targeting tumor antigens, leading to immune resistance [13]. By blocking these immune checkpoints with inhibitors, the “off” signal in the TME is disrupted, allowing T cells to resume their function of attacking and killing tumor cells. This treatment strategy has proven effective in various cancers, such as lung, bladder, and kidney, improving survival rates [196]. In OC, immune checkpoint inhibitors are also being explored in multiple clinical trials to improve disease management [197].

**Figure 7:**
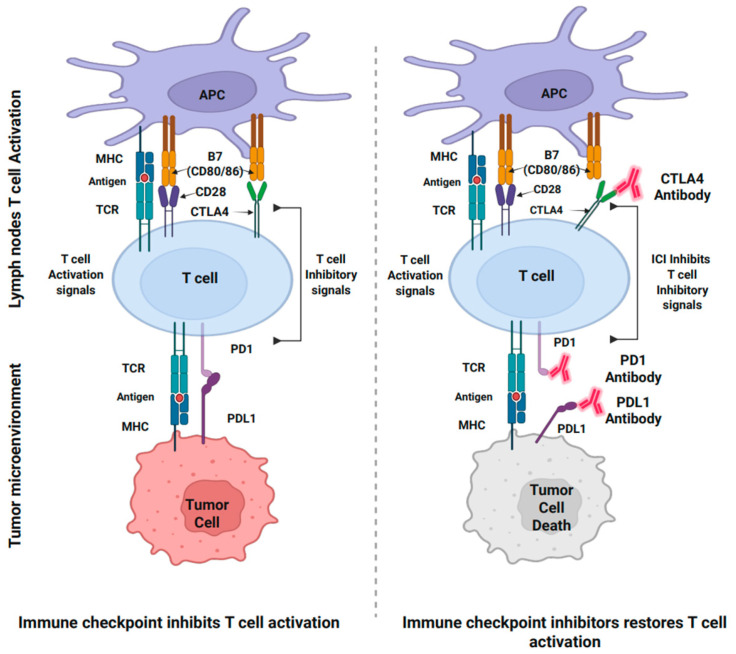
Immune checkpoints: LHS shows immune checkpoints inhibit T cell activation; In lymph nodes, APCs display antigen (red dots) on MHC molecules, which are recognized by TCRs. However, co-inhibitory molecules, such as CTLA4, outcompete CD28 for binding to B7, leading to the suppression of T cell activation. In TME, tumor cells also present antigens via MHC to TCRs, but PD1/PDL1 interaction further inhibits T cell activity, allowing tumor cells to evade immune destruction. RHS shows that checkpoint inhibitors restore T cell activation: Anti-CTLA4 antibodies block CTLA4, enabling stronger co-stimulation via CD28-B7 and T cell activation. Anti-PD1 or anti-PDL1 antibodies disrupt the PD1/PDL1 inhibitory axis. This restores T cell activation and cytotoxic function, enabling the immune system to recognize and kill tumor cells. LHS (left hand side); RHS (right hand side); APC (antigen-presenting cells); MHC (major histocompatibility complex); TCR (T cell receptor); CD8 (cluster of differentiation 28); CTLA4 (Cytotoxic T-lymphocyte antigen 4); PD1 (programmed death protein 1); PDL1 (programmed death protein ligand 1); ICI (immune checkpoint inhibitor).

Two immune checkpoints have been targeted in therapeutic cancer management, CTLA4 and PD1 (Figure 7) [196]. CTLA4, expressed on activated T cells, induces an immune-suppressive phenotype by competing with B7 (CD80) for binding to CD28, the activating receptor on T cells. In a Phase I clinical trial, ipilimumab, a CTLA4 inhibitor, demonstrated disease stability in three out of nine patients with stage IV OC [198]. Additionally, combination therapy with ipilimumab (a CTLA4 inhibitor) and nivolumab (a PD1 inhibitor) has shown improved patient survival compared to monotherapy. This combination extended PFS from 2 to 3.9 months and OS from 21.8 to 28.1 months [199]. 

PD1 is a receptor on T cells that binds to its ligand, PDL1, inhibiting T cell-mediated destruction of tumor cells. Blocking the PD1/PDL1 interaction allows T cells to effectively target and eliminate tumor cells. Anti-PD1/PDL1 therapies have shown promising results in various clinical trials [196]. For example, in a Phase II OC trial, the combination of nivolumab and bevacizumab (a VEGF angiogenesis inhibitor) resulted in a median PFS of 12.1 months for platinum-sensitive patients and 7.7 months for platinum-resistant OC patients [200]. The targeted inhibition of immune checkpoints, such as CTLA4 and PD1, holds significant promise in managing OC, offering the potential to enhance anti-tumor immune responses and improve clinical outcomes for patients.

### 6.4. Combined Targeted and Immunotherapies in OC Potentiate Therapeutic Outcomes

The combination of targeted therapies and immunotherapies has emerged as a promising strategy for improving therapeutic outcomes in OC. Specifically, combining anti-PD1 compounds with PARP inhibitors has shown encouraging results in several clinical trials [201]. For example, a Phase II trial led by Drew et al. explored the combination of durvalumab (an anti-PD1 agent) with Olaparib (a PARP inhibitor) in OC patients who were platinum-sensitive and had a BRCA mutation [202]. The trial reported a significant response rate of 63%, with six patients achieving a complete response and fourteen experiencing a partial response. These results highlight the potential synergistic effect of combining checkpoint inhibitors with targeted therapies in OC. Additionally, the inclusion of low-dose oral cyclophosphamide (LDCy) alongside these therapies can further enhance drug efficacy. A Phase II trial showed that the combination of pembrolizumab, bevacizumab, and oral cyclophosphamide was well tolerated and provided clinical benefit in 95% of patients with recurrent ovarian cancer, with 25% of patients showing durable responses lasting over 12 months [203]. This combination therapy shows significant promise as a future treatment strategy for recurrent ovarian cancer.

### 6.5. Targeting IL6 in Combination with Immunotherapies

IL6 plays a complex, dual role in influencing the immune landscape of the OC-TME. It can either promote a highly active, immune-infiltrated “hot” TME or contribute to a highly immunosuppressive, “cold” TME [204]. OC is typically classified as a “cold” tumor, often due to IL6-induced immunosuppression through the activation of signaling pathways like STAT3 in cancer cells. This pathway promotes immune evasion by recruiting immunosuppressive cells, such as Tregs and MDSCs, which inhibit anti-tumor T cell response. As a result, there is reduced infiltration of anti-cancer immune cells, which diminishes the effectiveness of immune therapies, including checkpoint inhibitors [88,204]. Targeting IL6 with monoclonal antibodies and receptor blockers, such as tocilizumab and siltuximab, reduces IL6 levels and helps “heat up” the TME, thereby enhancing the effectiveness of checkpoint inhibitors like anti-PD1/PDL1. Future research should focus on understanding the spatiotemporal dynamics of IL6 during tumor progression and identifying patient subgroups that would benefit most from IL6-targeted therapies, especially in combination with immune checkpoint inhibitors.

## 7. IL6 in Liquid Biopsy: Screening, Diagnostic, Predictive, and Prognostic

IL6 is gaining recognition as a versatile biomarker in OC, with potential applications spanning early-stage detection, diagnosis at various stages, personalized treatment strategies, insights into disease progression, and predicting responses to various therapies. As a soluble biomarker, IL6 can be measured in liquid biopsies like blood and serum, which can be obtained through minimally invasive procedures. This makes it a convenient, non-invasive, and easily accessible approach, offering valuable insights for early screening, prompt diagnosis, outcome prediction, and prognosis assessment [36,87,133,205]. As a pro-inflammatory cytokine, IL6 is often elevated in OC patients, even before clinical symptoms appear. However, suboptimal levels of IL6 in the early stages reduce its sensitivity for the early detection of OC [87]. Additionally, its levels can fluctuate due to external factors such as stress, diet, acute inflammation, or benign and non-malignant conditions, potentially leading to false positives [206]. As a result, this lack of specificity in disease detection has prevented IL6 from being established as a sole biomarker for screening or diagnosis of OC. However, by combining IL6 detection with other biomarkers, it may be possible to effectively identify the specific stages and types of OC [87,133,207].

Several studies have highlighted the potential of IL6 in distinguishing between malignant and benign ovarian growths, with IL6 concentrations in the circulation typically being higher in OC patients than in those with benign ovarian growths [87]. This difference is even more pronounced in ascitic fluid, where IL6 levels in malignant growths are often over a thousand times higher compared to benign cysts [87]. Such elevated levels point to a severe inflammatory condition in OC malignancy. In fact, IL6 levels reflect the complexity and severity of tumor immunogenicity within the OC-TME, with higher levels being more common in advanced stages [87]. These elevated levels are also associated with clinical burden and survival outcomes [87,133,207,208].

Elevated levels of IL6 in OC underscore its potential to be included in OC detection [87]. For example, combining IL6 with other protein markers like TNFR2 could strongly indicate aggressive tumor activity in OC [40]. Additionally, microRNAs involved in IL6 signaling, particularly those regulating JAK/STAT3 or NFκB pathways, could enhance IL6’s ability to detect OC and differentiate it from benign growths [209].

The widely used CA125 marker, employed to monitor OC recurrence, has limitations in sensitivity and specificity for diagnosing OC, as its levels can be elevated in conditions other than OC [210]. However, IL6 could complement CA125, particularly when CA125 levels are borderline, aiding to distinguish malignant from borderline tumors [87,211]. Similarly, combining IL6 with HE4, another biomarker that detects OC recurrence, may improve OC screening or facilitate early tumor detection [87,211]. Furthermore, IL6 could be integrated into composite risk models such as the Risk of Ovarian Malignancy Algorithm (ROMA), which combines CA125, HE4, and menopausal status. This integration would position IL6 as a valuable candidate for enhancing early screening and improving the diagnostic accuracy of OC, especially when used in conjunction with other commonly employed biomarkers [211]. This biomarker panel may also be more pronounced in high-risk patients, including those with a strong family history, BRCA mutations, or homologous recombination repair defects [189]. Thus, regular minimally invasive monitoring of IL6 levels in the circulation of those high-risk individuals could enable earlier detection of OC compared to conventional imaging or more invasive methods. In this context, by measuring IL6 levels alongside other potential risk factors and cancer-related inflammatory mediators, IL6 could serve as an effective “screening and diagnostic” biomarker for OC.

IL6 is increasingly recognized as a potent prognostic biomarker in OC. Elevated IL6 levels are associated with poor prognosis, including shorter survival, shorter recurrence, and diminished response to therapy. High IL6 expression in OC correlates with tumor aggressiveness, driving immune responses such as metastasis and ascites development, as well as contributing to chemoresistance, especially in platinum-based therapies. Additionally, IL6 has been shown to create a “cold” tumor microenvironment in OC, making it less responsive to immunotherapy and leading to worse clinical outcomes [212]. Furthermore, combining IL6 levels with miRNA21 expression levels may aid in predicting tumor progression and chemoresistance [213]. Together, these factors highlight IL6’s critical role in modulating the OC-TME and influencing disease progression and clinical outcomes. Thus, regular monitoring of IL6 levels and targeting its inhibition throughout the disease course could provide valuable dynamic prognostic insights and potentially improve disease management.

Tumor-derived IL6 has been linked to thrombocytosis in OC. IL6 acts on hepatocytes to stimulate the release of thrombopoietin (TPO), which increases platelet production. Elevated platelet levels are associated with tumor proliferation and the development of venous thromboembolism (VTE), a life-threatening complication in OC patients. Thrombocytosis also correlates with poor survival outcomes in OC patients [214,215,216].

IL6 levels are closely linked to a patient’s response to various therapies, particularly chemotherapy and immunotherapy, making it a valuable “predictive biomarker” [36,212]. The cytokine supports the survival and regeneration of cancer stem cells, contributing to immune evasion by altering the OC-TME [212]. IL6 targeted therapies, such as IL6 blockers or IL6 receptor blockers that target the IL6/IL6R axis, have shown promising results in preclinical models of OC, sensitizing the tumors to chemotherapy [36]. IL6 levels have also been suggested as a predictor of how likely OC patients are to respond to chemotherapy, both with and without the addition of IL6-targeted therapies [217].

IL6 could be integrated into a broader biomarker panel to improve the prediction of therapeutic responses, particularly for combination treatments like chemotherapy and bevacizumab, a VEGF inhibitor [218]. Additionally, measuring IL6 alongside PDL1 could help identify patients who may benefit from combination therapies involving anti-IL6 therapy and immune checkpoint inhibitors [36,219]. By regularly monitoring IL6 levels, clinicians could better tailor treatments and use precision medicine to select patients most likely to benefit from specific therapeutic interventions.

When combined with other biomarkers, advanced models incorporating machine learning (ML), artificial intelligence (AI), and bioinformatics, in addition to the existing platforms, have the potential to transform the use of IL6 in screening, diagnosis, prognosis, and predictive biomarkers (Table 3) [220,221,222,223]. Through dynamic, real-time trend analysis and multimodal data integration, ML and AI can facilitate earlier screening by identifying high-risk patients before clinical symptoms appear, support risk stratification, predict overall survival (OS) and progression-free survival (PFS), and guide optimized therapeutic interventions [223,224,225].

**Table 3:** Machine learning and Artificial intelligence impact on Interleukin 6 for early, precise, and personalized Ovarian Cancer management.

Category	Application	Details	Examples/Methods
Screening [222]	Early Detection	AI analyzes longitudinal IL6 trends to flag high-risk asymptomatic patients.	Time series analysis, anomaly detection models.
	Risk Stratification	Combines IL6 with other biomarkers (e.g., CA-125, HE4, miRNAs) for better screening accuracy.	Multimodal data fusion, feature selection.
Diagnosis [223]	Disease Diagnosis	Differentiates malignant ovarian cancer from benign ovarian conditions using IL6 and additional biomarkers.	Classification models like Random Forest or Support Vector Machines (SVM).
	Stages Detection	Integrates IL6 with markers like CRP, miRNAs, and imaging data to enhance diagnostic accuracy of specific stages or types.	Neural networks, ensemble learning.
Prognosis [224,226]	Survival Prediction	Uses IL6 levels and trends with other markers to predict overall survival (OS), progression-free survival (PFS), and disease-free progression (DFP).	Survival analysis with Cox proportional hazards models augmented by ML.
	Risk Stratification	Identifies high-risk patients based on IL6 levels, tumor aggressiveness, and likelihood of recurrence.	Decision trees, clustering algorithms for subgroup analysis.
	Monitoring Disease Evolution	Tracks IL6 and related biomarkers over time to predict metastasis, ascites development, or resistance.	Real-time monitoring with recurrent neural networks (RNNs) or Long Short-Term Memory (LSTM) networks.
Predictive [224,225]	Therapy Response Prediction	Predicts patient responses to chemotherapy, immunotherapy, or IL6/IL6R blockers by analyzing signaling pathways and biomarker profiles.	Predictive analytics using gradient boosting or deep learning.
	Personalized Treatment Planning	Identifies patients most likely to benefit from targeted therapies by integrating IL6 data with genomic and transcriptomic profiles.	AI-driven precision medicine platforms, supervised learning models.
	Clinical Trial Optimization	Selects ideal candidates for trials of novel therapies targeting IL6-mediated pathways.	AI-based cohort selection and predictive modeling.

Validating IL6 as part of a screening algorithm will require large-scale clinical trials to assess its performance in both asymptomatic and symptomatic populations. This approach would allow for the evaluation of IL6’s sensitivity, specificity, and ability to detect ovarian cancer at earlier stages, particularly in high-risk individuals. Early detection could lead to timely interventions and improved patient outcomes. Table 3 below proposes how ML and AI can leverage IL6 levels for OC screening, diagnosis, prognosis, and prediction.

## 8. Conclusions

In conclusion, IL6 plays a pivotal role in shaping the inflammatory microenvironment of ovarian cancer, influencing both tumor biology and clinical outcomes. Through activating the STAT3 signaling pathway, IL6 promotes critical processes such as angiogenesis, epithelial-to-mesenchymal transition (EMT), and immune evasion, all contributing to tumor progression and therapeutic resistance. IL6’s impact on the immune landscape categorizes ovarian tumors as either “hot” or “cold”, with its levels driving immune suppression in “hot” tumors and stromal remodeling in “cold” tumors. Furthermore, IL6 is strongly associated with disease stage and histological subtypes, particularly in high-grade serous carcinoma, where elevated levels correlate with more aggressive disease and poor prognosis. IL6 serves as a valuable biomarker for diagnosing, predicting, and prognosticating ovarian cancer (OC), offering key insights into tumor behavior, treatment response, and patient survival. Early diagnosis, combined with the precise selection of treatment, plays a crucial role in enhancing the overall survival of OC patients.

## Data Availability

This study did not create or analyze new data; hence, data sharing is not applicable to this article.
